# Prospective study and proposal of an outcome predictive nomogram in a consecutive prospective series of differentiated thyroid cancer based on the new ATA risk categories and TNM

**DOI:** 10.3389/fendo.2023.1128963

**Published:** 2023-03-13

**Authors:** Giulia Sapuppo, Sonia Grasso, Guenda Di Benedetto, Antonino Belfiore, Gabriella Pellegriti

**Affiliations:** ^1^ Department of Clinical and Experimental Medicine, University of Catania, Garibaldi-Nesima Medical Center, Catania, Italy; ^2^ Researcher in Oncology, Department of Clinical and Experimental Medicine, University of Catania, Catania, Italy

**Keywords:** differentiated thyroid cancer, thyroid cancer outcome, thyroid cancer risk factors, persistent or recurrent disease, TNM staging system, risk stratification system TNM staging and ATA risk stratification systems

## Abstract

**Introduction:**

The personalized management of differentiated thyroid cancer (DTC) is currently based on the postoperative TNM staging system and the ATA risk stratification system (RSS), both updated in 2018 and 2015, respectively.

**Purpose:**

We aimed to evaluate the impact of the last two editions of TNM and ATA RSS in the prediction of persistent/recurrent disease in a large series of DTC patients.

**Patients and methods:**

Our prospective study included 451 patients undergone thyroidectomy for DTC. We classified the patients according to TNM (both VIII and VII ed.) and stratified them according to the ATA RSS (both 2015 and 2009). We then evaluated the response to the initial therapy after 12-18 months according to the ATA “ongoing” risk stratification, and analyzed the variables associated with persistent/recurrent disease by multivariate analysis.

**Results:**

The performance of the last two ATA RSSs was not significantly different. By staging patients according to the VIII or VII TNM editions, we found significant differences only in the distribution of patients with structural disease classified in stages III and IV. At multivariate analysis, only T-status and N-status were independently associated with persistent/recurrent disease. Overall, ATA RSSs and TNMs showed low predictive power in terms of persistent/recurrent disease (by Harrell’s test).

**Conclusions:**

In our series of DTC patients, the new ATA RSS as well as the VIII TNM staging provided no additional benefit compared to the previous editions. Moreover, the VIII TNM staging system may underestimate disease severity in patients with large and numerous lymph node metastases at diagnosis.

## 1 Introduction

Thyroid cancer (TC) is the most common endocrine malignancy, representing about 90% of cases. TC is the most rapidly increasing cancer in the United States, where its incidence increased by 211% in the years 1975-2013; however, due in part to the adoption of more conservative diagnostic criteria, the incidence rate declined by 2.5% per year from 2014 to 2018 (1). The mortality from TC is very low and the death rate increased slightly during 2008 to 2017 (0.6% per year) despite earlier diagnosis and better treatment. In recent years TC death rate appears fairly stable (1). The prognosis of differentiated thyroid cancer (DTC) is excellent with a 5-year survival rate of 99-100% for localized, 98% for regional and 53% for metastatic disease (2).

In 2015 the American Thyroid Association (ATA) guidelines for DTC management (3) introduced a new risk stratification system, which included some supplementary prognostic factors such as lymph node characteristics (number, size and extranodal extension), mutational status, and foci’s number of vascular invasion. However, as stated in the ATA guidelines “the incremental benefit of adding these specific prognostic variables to the 2009 initial risk stratification system has not been established” and the added value of this modification has not yet been validated.

The TNM classification has also been updated in 2018 to better predict DTC survival (4). As a result of the changes, the eighth edition of the TNM results in the downstaging of a significant percentage of patients to more accurately indicate the low specific risk of death. Currently, the appropriate clinical-therapeutic management of TC requires post-surgical TNM staging to predict survival, and the assessment of the risk of persistent/recurrent disease according to the ATA risk stratification system. During the follow-up this risk is also regularly re-evaluated according to the “ongoing risk stratification” (3) based on the measurement of thyroglobulin (Tg), thyroglobulin antibodies (TgAb), neck ultrasound, post-131-I WBS, other imaging evaluation as required.

As expected, a more personalized and accurate assessment of the risk of persistent or recurrent disease and death from TC has a significant impact on the initial treatment decision (extension of thyroid and/or lymph node surgery, need for radioactive iodine ablation/therapy, need for TSH suppressive therapy) and appropriate management strategies during short and long-term follow-up.

In the present study we prospectively evaluated, in a continuous series of 451 DTC patients, the variables associated with persistent/recurrent disease (biochemical and structural) and the predictive power of the different ATA risk categories and TNM staging systems. Our findings may help establishing a tailored treatment management based on tumor and patient characteristics.

## 2 Patients and methods

We studied a consecutive series of 451 DTC patients undergone thyroidectomy with or without lymphnode dissection from October 2017 to February 2020 and followed up at our thyroid outpatients’ clinic. The median follow-up was 20.5 (IQR 14.7-27.4) months.

We staged the tumors according to the 7th and 8 th TNM editions: T (the greatest size of the primary tumor) and N (regional lymphnodes metastases) through histology. We indicated N0 when all removed lymphnodes were negative or if there is no radiological or clinical evidence of lymphnode metastasis, N1a if only the central compartment (levels VI-VII) is involved and N1b if latero-cervical nodes (levels I–V) were positive. M (distant metastases) was evaluated according to the post-surgical 131I-whole body scan (WBS) and/or other imaging evidence.

Disease status was evaluated in all patients through neck ultrasound and Tg and TgAb determination after surgery and periodically during the follow-up (every 3-6 or 12 months).

After 12-18 months from the first evaluation, the response to initial therapy (surgery with or without iodine treatment) was assessed with neck ultrasound and both serum Tg and TgAb measurements, either basal or TSH stimulated (L-thyroxine, LT4withdrawal or rhTSH injection) in radioiodine treated patients. According to their response to the initial therapy (ongoing stratification) patients were re-classified as excellent response (ER), indeterminate (IR), biochemical incomplete (BIR) or structural incomplete response (SIR).

Subsequent follow-up was modulated based on the initial risk evaluation and the first treatment response.

The level of TSH-suppression was based according to the ATA guidelines: substitutive LT4 therapy in low-risk patients without evidence of disease; mild TSH suppression (0.1-0.4 mU/L) in intermediate risk patients or biochemical disease; complete suppression (< 0.1 mU/L) in high risk patients and/or if structural disease.

All patients with the evidence of persistent or recurrent disease underwent additional morphological exams (computed assisted tomography (CT scan), magnetic resonance imaging, bone scan, positron emission tomography). If patients were not cured, further treatments such as radioiodine treatment therapy, other surgeries or different therapies) were brought off.

## 3 Statistical analysis

Categorical variables were expressed as frequencies and percentages; quantitative normally distributed ones as mean ± standard deviation (SD) and non-normally distributed ones as median with interquartile range (IQR). The normality was verified through the Kolmogorov-Smirnov test.

The Chi-square test with Yates’s correction or Fisher’s test were used to analyze the categorical variables. Multivariate analysis was outbrought off through the logistic regression including only significant variables for recurrent/persistent disease at univariate analysis.

The predictive power of the different ATA risk categories and TNM staging systems at short-term re-evaluation was assessed by the Harrell’s C concordance index (C-index).

A nomogram was implemented based on the parameters that resulted significantly associated with the risk of recurrent/persistent disease at the multivariate logistic regression analysis, with a risk score for predicting the probability of persistent and recurrent disease.

A p-value <0.05 was considered statistically significant for all analyses. Data analysis was performed using the Stata software version 16.

## 4 Results

Clinical and histopathological characteristics are shown in **Table 1**. Most patients were females with a F/M ratio of 2.6/1.0. Median age at diagnosis was 47.5 yrs (IQR 36.9-57.8). Histotype was papillary in almost all cases (96%). 14.2% of patients had an aggressive PTC variant (diffuse sclerosing, tall cell, insular or columnar).

**Table 1:** Clinical and histopathological characteristics of the 451 DTC patients.

	n.	(%)
Patients (n.)	451	
Follow-up median (IQR) (months)*	20.5 (14.7-27.4)	
Age median (IQR) (y)	47.5 (36.9-57.8)	
Gender		
F/M (ratio)	324/127 (2.6/1)	
Histotypes		
Papillary	433	96.0
Follicular	12	2.7
Papillary-follicular	4	0.9
Poorly-differentiated	2	0.4
M1	11	2.4
Minimal extrathyroid extension (ETE)	97	21.5
Multifocality	176	39.0

* Follow-up minimum 6 months.

Eleven patients (2.4%) had distant metastases at diagnosis (10 lung metastases and 1 with lung and bone metastases).

Minimal extrathyroid extension was present in 21.5% and multifocality in 39.0% of patients.

Most patients fell into the T1 and T2 categories, especially with the new TNM staging system; 26.8% of patients had central (N1a) and 13.7% latero-cervical lymphnode metastases (N1b).

Two hundred seventy-seven (61.4%) patients were treated with 131I with different activities: 85 patients with 30 mCi, 186 patients with 100 mCi, 3 patients with 50 mCi, 1 patient with 70 mCi, 1 patient with 150 mCi e another 1 with 200 mCi. 156 patients were treated after L-T4 withdrawal and 121 after rhTSH administration. Six (1.3%) patients required another surgical treatment in the neck and 6 (1.3%) patients underwent to a second 131I treatment after about 12 months from the first.

### 4.1 TNM staging system and risk stratification system

TNM, staging (VII and VIII editions) and Risk stratification classifications are shown in **Table 2**.

**Table 2:** TNM, staging (VII and VIII editions) and risk categories (2009 and 2015) of the 451 DTC patients.

		n.	(%)			n.	(%)
TNM (VII ed.)				TNM (VIII ed.)			
T status (T)				T status (T)			
T1a		166	36.8	T1a		206	45.7
T1b		105	23.3	T1b		143	31.7
T2		56	12.4	T2		73	16.2
T3		113	25.1	T3a		5	1.1
T4a		5	1.1	T3b		13	2.9
Tx		6	1.3	T4a		5	1.1
				Tx		6	1.3
N status (N)				N status (N)			
N0		54	12.0	N0a		124	27.5
N1a		121	26.8	N0b		144	31.9
N1b		62	13.7	N1a		121	26.8
Nx		214	47.5	N1b		62	13.7
Staging (VII ed.)				Staging (VIII ed.)			
<45	I	202	44.8	<55	I	298	66.1
	II	4	0.9		II	10	2.2
≥45	I	122	27.1	≥55	I	92	20.4
	II	19	4.2		II	39	8.6
	III	62	13.7		III	5	1.1
	IVA	34	7.5		IVA	0	
	IVB	0			IVB	7	1.6
	IVC	8	1.8			
Risk categories at first evaluation 2009 guidelines				Risk categories at first evaluation 2015 guidelines			
Low		193	42.8	Low		205	45.5
Intermediate		247	54.8	Intermediate		231	51.2
High		11	2.4	High		15	3.3

Applying VII TNM staging system 60% tumors were T1a and T1b,instead applying VIII TNM more than ¾ of the patients (77.4%) fall into this category. This different percentage depends mainly on the removal of minimal extrathyroid extension from T3 classification.

Regarding the lymph node status, applying VII TNM staging 12.0% patients were N0 47.5% Nx; instead applying VIII TNM staging almost 60% of cases were N0a or N0b (268, 59.4%) (due to the removal of Nx in the new TNM system). Thesame percentages were in N1a and N1b classes.

Six patients presented latero-cervical metastases without involvement of the central compartment (skip metastases).

**Figure 1:**
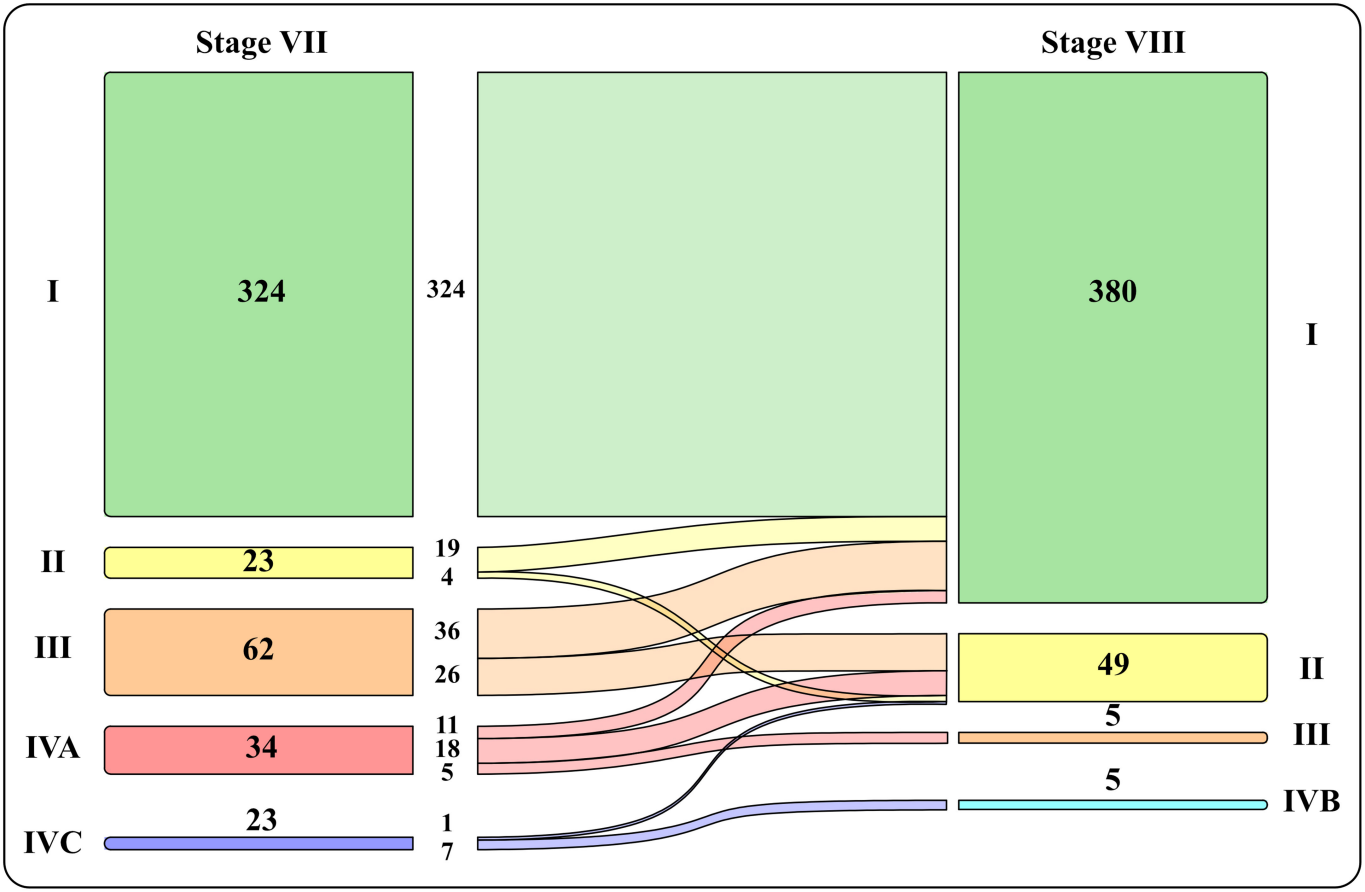
Graphic representation of patients’ distribution into the four stages with VII vs VII TNM staging system.

Patients were also staged comparing VII vs VII TNM staging system (**Figure 1**): most patients fell into stage I using both classifications, respectively 71.9% vs 86.5%.Using VIII TNM edition there was a significative downstaging in all categories (about 30%), mostly from stage III and IVA into stage I and II.

At initial evaluation, patients were subdivided into three different risk categories with few variations in percentage due mainly to the lymph node number and size categorization using 2009 or 2015 ATA risk stratification: 42.8% and 45.5% low, 54.8% and 51.2% intermediate and 2.4% and 3.3% high risk according to respectively 2009 and 2015 ATA risk system.

### 4.2 Response to initial therapy, 12-18 months after initial treatment, in all patients

After initial treatment, 63.9% of patients presented with excellent response. However, 35 patients (not ablated) had basal Tg between 0.2 and 1 ng/mL, stable and compatible with small thyroid remnant. After 12-18 months from initial treatment, just over a third of patients (36.1%) were not cured.

In particular 82 patients presented an indeterminate response (68 patients had indeterminate Tg or TgAb and 14 patients non-specific finding at neck ultrasound or CTscan). Five patients had biochemical incomplete response and 76 patients structural incomplete response (52 had lymph node metastases, almost all small in number and size; 10 lung metastases; 1 only bone metastases and 5 lung and bone metastases, 5 lung and LN metastases, 2 lung, bone and local disease).

#### 4.2.1 Response to initial therapy according to ATA risk categories

**Figure 2:**
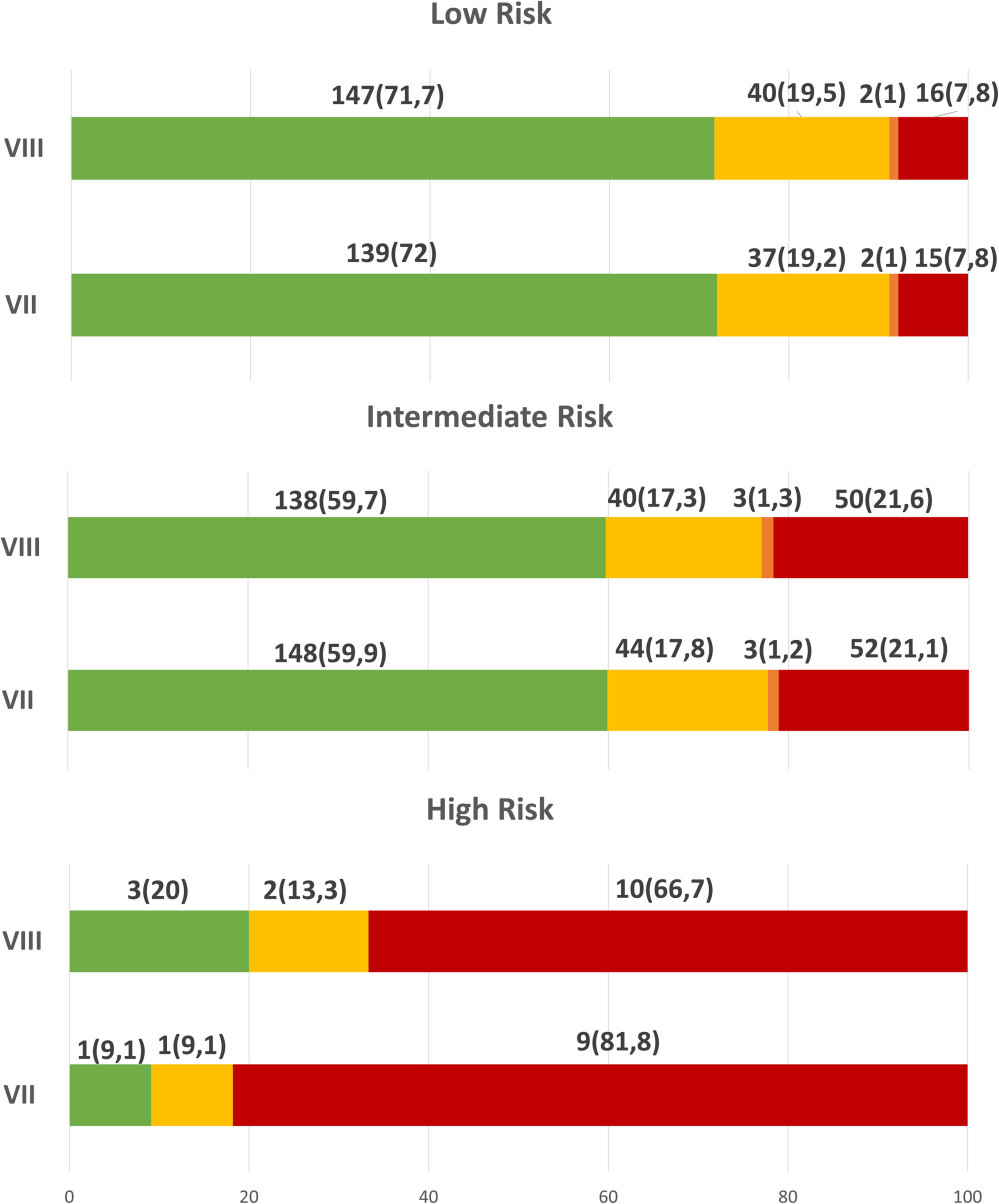
Response to initial therapy, 12-18 months after initial treatment, according to ATA risk categories.

As expected, the percentage of patients with an excellent response decreased through risk categories, being less frequent in intermediate- and mostly high-risk patients with both ATA risk categories (**Figure 2**).

In low risk patients, no significant difference was found in both TNM: approximately 72% had an ER, 19% IR, 1% BIR and 7.8% SIR.

Also in intermediate risk patients, no significant difference was found: approximately 60% ER, 17.5% IR, 1% BIR and 21% SIR.

Instead in high risk patients, 20% vs 9.1% had ER, 13.3% vs 9.1% an IR and 66.7% vs 81.8% SIR, respectively using 2009 and 2015 ATA risk stratification.

The results did not change after analysing classic PTC subtype and all DTC separately.

### 4.3 Structural disease after 12-18 months after initial treatment according to VII vs VIII TNM staging

**Figure 3:**
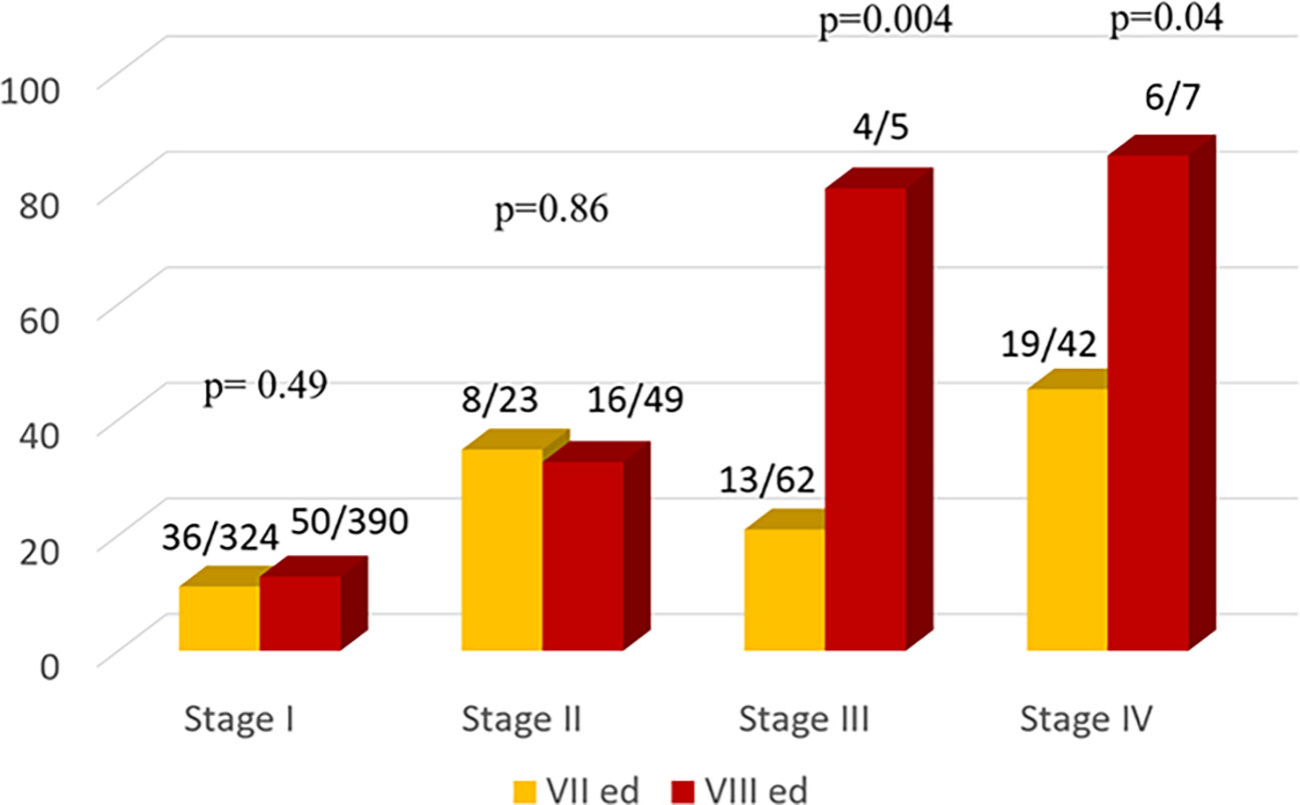
Comparison of the Number of Patients with structural disease according to the Seventh (yellow bars) and Eighth (red bars) TNM Editions.

As expected, the percentage of patients with structural disease increases through the stages mostly using VIII TNM ed. (**Figure 3**).

Comparing the two TNM editions, a similar percentage of disease was observed in stage I and II both using VII or VIII TNM edition instead a significant difference in stage III and IV, respectively p=0.004 and 0.04.

### 4.4 Predictors of persistent/recurrent disease at 12-18 months after first treatment

Risk factors of persistent disease (either morphologic or biochemical) at last disease assessment are presented in **Table 3**.

**Table 3:** Risk factors of disease (biochemical and structural) at 12-18 months after primary treatment.

Variable	Biochemical and structural disease	Univariate analysis OR [95%CI]	p	Multivariate analysis OR [95%CI]	p
**Age** - < 55 years - ≥ 55 years	105/308 (34.1) 58/143 (40.6)	1.32 (0.88-1.99)	0,18		
**Gender** - Female - Male	112/324 (34.6) 51/127 (40.2)	1.27 (0.83-1.94)	0.27		
**Lymph node surgery at primary treatment** **-** Performed - Not performed	117/307 (38.1) 46/144 (31.9)	1.31 (0.86-2.0)	0.20		
**Aggressive histology** - No - Yes	139/387 (35.9) 24/64 (37.5)	1.07 (0.62-1.85)	0.80		
**T status** - T1a - T1b - T2 - T3a - T3b - T4a	61/206 (29.6) 49/143 (34.3) 38/73 (52.1) 0/5 8/13 (61.5) 3/5 (60.0)	1.24 (0.78-1.96) 2.58 (1.49-4.46) 3.80 (1.20-12.09) 3.57 (0.58-21.88)	0.36 <0.001 0.01 0.14	0.99 (0.61-1.61) 2.11 (1.19-3.74) 2.54 (0.77-8.35) 2.96 (0.48-18.46)	0.98 0.009 0.12 0.24
**Multifocal** - No - Yes	92/274 (33.6) 70/176 (39.8)	1.31 (0.88-1.93)	0.18		
**Extra thyroidal invasion** - No - minimal	123/355 (34.6) 40/96 (41.7)	1.35 (0.85-2.14)	0.20		
**Lymph node metastases** - Absent -Present	77/268 (28.7) 86/183 (47.0)	2.20 (1.48-3.26)	<0.0001	Not included	
**Number of N1 at primary surgery** - N0a/N0b - ≤5 N1 - >5 N1	77/268 (28.7) 55/135 (40.7) 31/48 (64.6)	1.71 (1.10-2.65) 4.52 (2.37-8.65)	0.016 0.0000	Not included	
**Location of N1 at primary surgery** - N0a/N0b - N1a - N1b	77/268 (28.7) 49/121 (40.5) 37/62 (59.7)	1.69 (1.08-2.65) 3.67 (2.07-6.51)	0.02 0.0000	1.40 (0.87-2.25) 3.07 (1.68-5.62)	0.16 <0.001
**ATA risk stratification** - Low - Intermediate - High	58/205 (28.3) 93/231 (40.3) 12/15 (80.0)	1.71 (1.14-2.55) 10.14 (2.76-37.24)	0.008 0.0000	Not included	
**Radioiodine treatment** - Not performed - Performed	37/174 (21.3) 126/277 (45.5)	3.09 (2.00-4.77)	0.0000	Not included	

At univariate analysis, the factors associated to the presence of disease were: T status, the presence of lymph node metastasis, both in the central compartment and in lateral compartments (N1a and N1b status), the presence of more than five lymph node metastases, ATA risk intermediate or high and radioiodine treatment therapy.

At multivariate analysis, both T status and lateral lymph node metastasis were patient features independently predicting persistent/recurrent disease (higher O.R. for N1b= 3.07, p<0.001).

Taking into account only structural incomplete response (SIR) at univariate analysis, the factors associated to the presence of persistent disease are, beyond the same risk factors of above, were male gender and multifocality. At multivariate analysis T status and lateral-lymph node were independent predictors of disease (**Table 4**).

**Table 4:** Risk factors of structural disease at the 12-18 months assessment after primary treatment.

Variable	Structural disease n.76	Univariate analysis OR [95%CI]	p	Multivariate analysis OR [95%CI]	p
**Age** - < 55 years - ≥ 55 years	51/308 (16.6) 25/143 (17.5)	1.07 (0.63-1.81)	0.80		
**Gender** - Female - Male	46/324 (14.2) 30/127 (23.6)	1.87 (1.12-3.13)	0.016	1.63 (0.92-2.90)	0.09
**Lymph node surgery** - Performed - Not performed	57/307 (18.6) 19/144 (13.2)	0.67 (0.38-1.17)	0.155		
**Aggressive histology** - No - Yes	63/387 (16.3) 13/64 (20.3)	1.31 (0.67-2.55)	0.42		
**T status** - T1a - T1b - T2 - T3a - T3b - T4a	23/206 (11.2) 21/143 (14.7) 21/73 (28.8) 0/5 (0.0) 5/13 (38.5) 3/5 (60.0)	1.37 (0.73-2.58) 3.21 (1.65-6.26) 4.97 (1.50-16.49) 11.93 (1.89-75.22)	0.33 <0.001 0.004 0.001	1.12 (0.58-2.18) 2.76 (1.37-5.56) 3.18 (0.82-12.34) 10.85 (1.60-73.52)	0.73 0.005 0.09 0.01
**Multifocal** - No - Yes	38/274 (13.9) 37/176 (21.0)	1.65 (1.00-2.72)	0.04	1.16 (0.66-2.04)	0.60
**Extra thyroidal invasion** - No - minimal	54/355 (15.2) 22/96 (22.9)	1.66 (0.95-2.89)	0.07		
**Lymph node metastases** - Absent - Present	30/268 (11.2) 46/183 (25.1)	2.66 (1.61-4.42)	<0.001	Not included	
**Number of N1 at primary surgery** **-** N0a/N0b - ≤5 N1 - >5 N1	30/268 (11.2) 53/130 (40.8) 31/48 (64.6)	5.46 (3.26-9.15) 14.47 (7.16-29.22)	<0.001 <0.001	Not included	
**Location of N1 at primary surgery** - N0a/N0b - N1a - N1b	30/268 (11.2) 28/121 (23.1) 16/62 (25.8)	2.39 (1.35-4.22) 2.76 (1.39-5.47)	0.002 0.002	1.59 (0.84-3.03) 4.04 (2.00-8.13)	0.15 <0.001
**ATA risk stratification** - Low - Intermediate - High	16/205 (7.8) 50/231 (21.6) 10/15 (66.7)	3.26 (1.79-5.94) 23.63 (7.20-77.55)	<0.001 <0.001	Not included	
**Radioiodine treatment** - Not performed - Performed	8/174 (4.6) 68/277 (24.5)	6.75 (3.16-14.44)	<0.001	Not included	

Since the multivariate analysis showed that lymph node metastases have a significant impact to predict persistent/recurrent disease, LN characteristics (localization and number) have been investigated in detail.

As expected not only the presence of positive LN (N1a + N1b vs N0/Nx), but also the number of nodal metastases and their location (N1b vs N1a) were relevant risk factors for persistent/recurrent disease persistence at 12-18 months after first treatment.

Persistent disease was significantly higher in N1 vs N0a/N0b patients (47% vs 28.7%, p<0.001).

Analyzing the number of nodal metastases, using the cut off of 5 positive nodes, the likelihood of persistent disease was higher for patients with > 5 vs ≤ 5 metastatic lymph nodes or negative lymph node (respectively 64.6%, 40.8% and 28.7%) (p<0.001) (**Table 3**).

As for lymph node location (N1a or N1b), the frequency of disease at 12-18 months after first treatment progressively increased from 28.7% in N0a/N0b to 40.5% in N1a and to 59.7% in N1b (OR 1.69 and 3.67 and p=0.02 and <0.001, respectively) (**Table 3**).

Analyzing the number and location of nodal metastases, for 121 N1a patients, the probability of persistent disease was higher when more than 5 lymph nodes were involved (7/13 cases, 53.8%) compared with ≤5 lymph nodes involved (42/108, 38.9%); this difference was not statistically significant (p=0.30).

For 62 N1b patients, the probability of persistent disease was sensibly higher when more than 5 lymph nodes were involved (24/35, 68.5%) compared with ≤ 5 lymph nodes involved (13/27, 48.1%); also this difference was not statistically significant (p=0.10).

### 4.5 Predictive power of risk stratification system (ATA 2009 vs. ATA 2015) and TNM staging system (VII vs. VIII edition)

To evaluate the accuracy of the prediction of the different ATA risk stratification systems (2009 vs. 2015) we calculated the Harrell C concordance index (C-index) considering the disease events at 18-24 months of re-evaluation. We found no differences in predictive power between the ATA 2009 and ATA 2015 criteria for biochemical and structural disease (C-index = 0.576 and 0.574, respectively for ATA 2009 and ATA 2015).

This still held true when only patients with structural disease were considered (C-index = 0.656 and 0.657, respectively for ATA 2009 and ATA 2015).

Similarly, the VII and the VIII TNM staging system resulted to have a similar and low predictive power (C-index = 0.560 vs 0.570, for the VII and the VIII edition, respectively).

### 4.6 Development of a nomogram to predict persistent/recurrent disease

**Figure 4:**
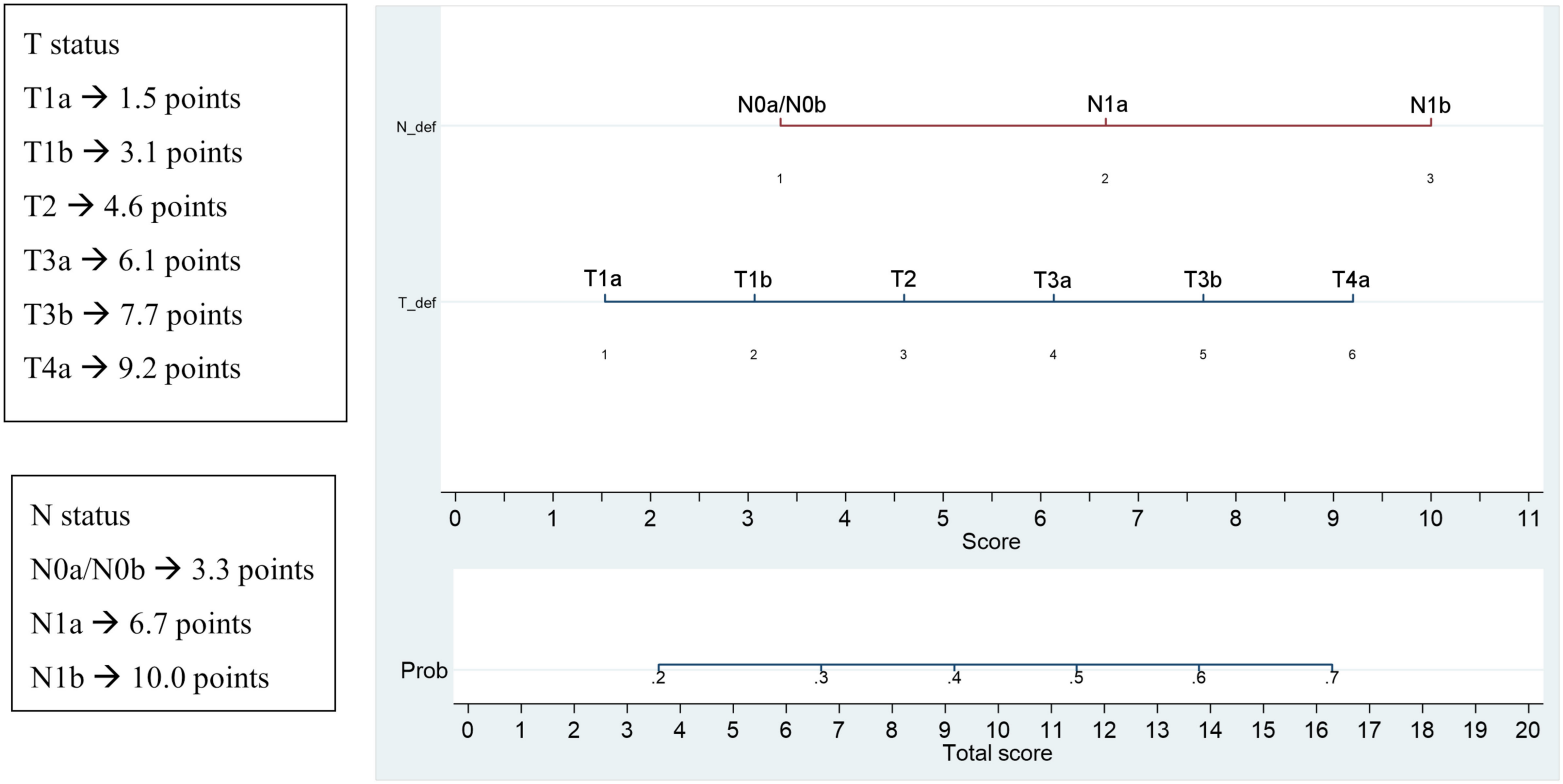
Significant parameters at multivariate and corresponding prognostic points and nomogram for the prediction of persistent/recurrent disease on the basis of clinical and histological characteristics.

A nomogram incorporating all the significant parameters was constructed based on the multivariate logistic model identified in **Table 3**. For each parameter we obtained a corresponding prognostic points as shown in **Figure 4**. The point values for all predictor variables were summed to reach a total score. This value was plotted on the total score axis and a vertical line drawn from this axis straight up that indicates the patient’s probability of persistent/recurrent disease at re-evaluation after the first therapy.

## 5 Discussion

Differentiated thyroid cancer (DTC) is the most common endocrine malignancy (5). The most frequent histotype (over 85%) is papillary (PTC). DTC incidence was quite stable until 1990s and in recent decades has rapidly grown, more than any other cancer (6), mainly due low-risk thyroid cancer increasing (https://www.cancer.org/research/cancer-facts-statistics/all-cancer-facts-figures/cancer-facts-figures-2022.html) (1).

DTC’s prognosis is generally excellent., but in some patients tumors are aggressive with poor outcome.During 2008 to 2017 (0.6% per year) TC’s death rate increased slightly but appears to have stabilized in recent years.

It is not known if the incrising incidence of PTC is true or whether it is due to the overdiagnosis of indolent PTCs.

Finding the variables identifying these tumors is a major issue to decide the appropriate management.

The American Thryoid Association (3) introduced a new risk stratification system with additional prognostic variables. Moreover, to better predict DTC survival, also the TNM classification was updated in 2018. A significant number of patients were downstaged by the 8th edition (TNM-8) into lower stages to more accurately reflect the low risk of dying, but underestimating the risk of persistent/recurrent disease and death in some patients due to the fact that all young patients without distant metastases fall into stage I.

The changed risk stratification and TNM staging have a significant consequences on the earliest therapeutic judgment and subsequent follow‐up management.

Concerning the risk stratification, different data have been published. In Steinschneider et al. data (7) approximately 70% of patients were low risk, 25% intermediate and 5.2% high risk patients. Instead, in our data showed fewer patients in low category (45%) and higher in intermediate risk (51%) than in literature with no significant difference between 2009 and 2015 risk stratification.

Regarding the staging, a large proportion of patients were downstaged in the 8th edition (30-40%) vs the 7^th^ one, mostly due to the increasing of the age cut-off to 55 years, the down-classification of T3 disease, and the overall downstaging of lymphnode metastases (4, 7–9), with a minimal impact on the expected 10-year disease-specific survival despite the large proportion of shifted patients to stage I and II. Kim et al. (4) found that 41% of patiens were downstaged and inevitably more patients with recurrences or deaths were found in the lower stages: 17% of patients downstaged from stage III to II had recurrent disease, 25% and 13.6% died in the group downstaged respectively from stage IV to III and from stage IV to II.

Also our data shows an important downstaging (about 30%) mostly from staging III and IVA to I, II and III. In particular the downstaging concerned 82.6% of stage II (into stage I), 100% of stage III (into stage I and II) and 100% of stage IV (into stage I, II and III).

Evaluating the different response at initial therapy (dynamic or ongoing risk stratification), a paper published 10 years ago by Tuttle et al. (10) found an ER in 86% of low risk patients, in 57% of intermediate-risk and in 14% in the high-risk. In 11% of low-risk patients, 22% in intermediate-risk and 14% in high-risk had BIR and 3% of patients in the low-risk, 21% in the intermediate-risk, and 72% in the high-risk had SIR.

Another paper evaluating 441 patients (7) showed that the proportion of intermediate/high-risk patients in stages I–II increased considerably according to TNM-8 and that patients downstaged in stage II with TNM-8 had more lymphnode metastases, surgeries, disease persistence and an increased disease-specific mortality (non-significant) vs to TNM-7. They found a similar rates of persistent and recurrent disease in stage I in both TNM editions, but higher in stages II (p = 0.05) and III (p = 0.03) in TNM-8 vs TNM-7Therefore the new TNM guaranteed a more accurate system to assess mortality and persistence disease but that the severity, mainly in in stage II patients or in the 45–55-year old group, should not be underestimated as a result of the important downstaging of some particular groups of patients.

In the present study, after initial treatment, 63.9% of patients presented ER and 36.1% patients were not cured, of which half presented an IR, a little less cases SIR and few patients BIR.

Our data are different compared to previous data mostly for lower percentage of the ER in low risk patients (71.7%), due to an higher number of patients IR (19.5% of low risk patients, 17.3% in intermediate-risk patients and in 13.3% of the high-risk patients) and very lower percentage of patients with BIR (1% in the low-risk, 1.3% in the intermediate-risk and 0% in the high-risk group); regarding SIR the rate are similar to the other paper (in 7.8% in the low-risk, 21.6% in the intermediate-risk, and 66.7% in the high-risk group).

Moreover in our patients the rates of structural disease in stage I and II was similar in both editions and it was significantly higher in stages III (p = 0.004) and IV (p = 0.04) in VIII compared to VII edition.

Another issue still to be validated is the timing of the re-evaluation. Based on our data, it seems that the restaging at 12-18 months could be too early as many patients with indeterminate response could change into excellent response (for example for TgAb still positive but stable or declining). This hypothesis will be evaluated by extending the follow-up.

Concerning the risk factors, most reports show that age, gender, aggressive variants, tumor size, lymphnode metastases are the most important predictors of outcome (3).

In a recent paper in 2020, Shin et al. (11) found that at at multivariate analysis tumour size > 4 cm, multifocality and nodal factors were the independent factors of recurrence free survival.

In our data only tumor size and lymph node metastases independently predicted short term outcome, instead the other risk factors were not statistically significant. In fact, tumor size is an independent risk factors from T2 up to T4a both for recurrent/persistent disease (biochemical and structural) and for only structural disease.

In a retrospective analysis of 574 patients with PTC, Tran et al. (12) found that tumor size predicted recurrence-free survival on multivariate analysis and Pellegriti et al. (13) showed that tumor size (≤1.0 cm versus. 1.1–1.5 cm) was not predictive of recurrence. Nguyen et al. (14) showed that, in SEER database, the10-year relative survival rates for tumors sized 1.5 cm or larger and tumors less than 1.5 cm were 95.4% and 99.8%, respectively.

Regarding lymph node metastases, their clinical relevance in PTC has been a debated matter for decades (15, 16). To date, the LN metastases impact at diagnosis on recurrence’s risk is well documented in many papers, including a recent study of our group (17, 18). For many years, however, only neck lymhpnode metastases, without other specified variables, was evaluated as a PTC prognostic factor.

The impact of neck metastatic LN on PTC risk stratification was better defined in 2015 ATA guidelines, in which additional informations, as the number, size or extranodal extension of metastatic lymhpnodes, were included in the evaluation. To date, these additional characteristics have not been validated and their relevance in defining the recurrence risk has yet to be quantified.

In our series the positivity of lymph node metastases, the number (≤5 or >5) and the location (N1a and N1b) are effective predictors of the outcome of the patients at 12-18 months after the first treatment, both for recurrent/persistent disease (biochemical and structural) and for only structural disease.

Data on the location of metastases, N1a or N1b, were judged insufficient to include this information in the clinicopathologic variables to estimate the risk in PTC (3) (recommendation #48, [B20], paragraph 1, line 16).

In a recent publication of our group (19), N1b worsened the prognosis and may be related to the appearance of distant metastases, which are considered the best surrogate index for cancer-specific death.

N1b status could be a marker of more aggressive or more advanced disease at diagnosis in PTC patients and associated with other ones (as molecular alterations) of cancer aggressiveness.

Concerning the mETE, its role on disease specific survival (DSS) and on overall survival (OS) have been evaluated by several studies but it’s still consider a controversial prognostic factor. Some authors (19–22) showed similar clinical outcomes.However, Castagna et al. (23) showed poorer outcome, in term of persistent/recurrent structural disease and tumor-related death in patients with mETE vs tumors >1.5 cm without extrathyroid extension (11.8% vs. 5.1%),concluding that only small mETE cancers should be consider at low-risk.

Recently, an expansion of TNM-8 has been published (Telescoping) to test he subcategories, according to the mETE, to get a better estimate of the prognosis and to plan the follow-up management.In the next few years, the impact of mETE for each tumor class will be available.

Comparing disease specific survival (DSS) between TNM VII vs VIII ed, Tam et al. (20) concluded that DSS in both TNM editions is similar, although through the updated TNM-8 the 10-year DSS appears more proper between stages. For stages I–IV with TNM-7 the 10-year DSS ranged from 100% to 82.6% (p < 0.001) and the 10-year OS from 95.8% to 59.7% while with TNM-8 from 99.8% to 71.9% (p < 0.001) and from 94.3% to 34.6%, respectively.

Contrasting, results from Chung et al. (21), analyzing a large series of 3,176 DTC patients, and Jeon et al. (22), investigating the predictive capability of DSS of TNM‐8 compared to TNM‐7 in 1,613 DTC patients, showed that TNM-8 has a higher power to differentiate patients in each stage and to predict also the DSS.

Although therefore with the TNM-8 an improved assignment of patients at high risk of dying from DTC into more advanced stages of disease seems evident, on the other hand, leads to the erroneuous belief that the disease is less aggressive. Nearly 50% of the cancer-related deaths are involve patients in stages I-II, compared to none with the TNM-7 (7). Having thyroid cancer a very low mortality, in some patients the risk of death is not always related to recurrence’s risk.

Recently, several studies showed a better predictability for the new TNM (6, 24). A more accurate survival predictions is suggested when TNM-8 is applied, due to the downstaging of a significant number of patients (about 30%).

In our data, being the median follow-up of 20.5 months, mortality was not assessed so an analysis evaluating the presence of distant metastases, good surrogate for predicting mortality, was carried out. The C-Harrel test to evaluated the power to predict disease after short follow-up found no difference using VII and VIII TNM editions (both for biochemical and structural disease and also for structural disease).

Lastly, generally, cancer nomograms are prediction tools to assess the risk based on specific patient’s and tumor’s characteristics and to predict the likely outcomes of different therapies. By integrating different prognostic variables, the nomogramhas the ability to create an individual numerical probability of a clinical event, useful to improve disease prognostication and therefore a personalized follow-up.

In literature, recently, several nomograms for prediction the risk of death from thyroid cancer have been proposed (23, 25). In 2016 Lang et al. (24) validated a nomogram for PTC patients with an excellent discriminating skill and accuracy in predicting 10-years-disease-specific death and recurrence. In 2017 two nomograms were proposed (26), one for regional recurrence-free survival and one for distant recurrence-free survival prediction with a C-index of 0.72 and 0.83, respectively.

Also the nomogram elaborated by our data could be useful to plan an individualized follow-up for each patient based on the score obtained from the risk calculation. The limit of this system is the lack of external validation

In conclusion, although the new TNM-8 compared to the previous TNM edition would seem to better discriminate the disease-specific death, in some patients (as N1b at diagnosis, mainly if numerous and large) could underestimate the severity of disease due to the significant downstaging and bring to a non-negligible treatment burden. In fact while in the TNM-7 N1b patients were included in advanced risk categories, the new TNM-VIII does not discriminate the death rate according to the lymphnode location, downstaging some patients (particularly old patients and/or N1b) decreasing the discriminating ability for the few patients with a negative outcome, despite categorized as stage II. Therefore, a careful follow-up is needed for downstaged patients.

Further prospective studies are needed to better define the real effectiveness of the 2015 ATA risk stratification system and the VIII TNM staging system.

## Data availability statement

The raw data supporting the conclusions of this article will be made available by the authors, without undue reservation.

## Ethics statement

The studies involving human participants were reviewed and approved by Comitato Etico Catania 2 A.O. Garibaldi in Catania Piazza Santa Maria del Gesù, 5 95124 Catania. The patients/participants provided their written informed consent to participate in this study.

## Author contributions

I) Conception and design: GS and GP. (II) Collection and assembly of data: GS, GD, MR, AB and GP. (III) Data analysis and interpretation: GS and GP. (IV) Manuscript writing: GS and GP. (V) Final approval of manuscript: All authors. All authors contributed to the article and approved the submitted version.
